# Ocular toxoplasmic scar: a rare clinical image of an immunocompetent patient

**DOI:** 10.11604/pamj.2023.45.2.39808

**Published:** 2023-05-02

**Authors:** Tanvi Guru, Sachin Daigavane

**Affiliations:** 1 Department of Medicine, Jawaharlal Nehru Medical College, Datta Meghe Institute of Higher Education and Research, Sawangi (Meghe), Wardha, Maharashtra, India,; 2 Department of Ophthalmology, Jawaharlal Nehru Medical College, Datta Meghe Institute of Higher Education and Research, Sawangi (Meghe), Wardha, Maharashtra, India

**Keywords:** Ocular toxoplasmosis, immunocompetent, chorioretinal scar, fundus examination

## Image in medicine

A thirty-six-year-old male patient came with complaints of diminution of vision in the right eye for 10 years. He did not give a history of ocular trauma, redness or pain in that eye, seizures or any previous treatment. He is a non-vegetarian. There is no history of contact with cats. Clinically, the anterior segment examination of both eyes was normal. His best corrected visual acuity (BCVA) in the right eye was 6/60, and his left eye was 6/9, and intraocular pressure in both eyes was within normal limits. Posterior segment evaluation by direct ophthalmoscopy revealed a chorioretinal scar in the right macular area, fundus photograph showing a chorioretinal scar in the right macular area, which was secondary to toxoplasmosis characterised by the presence of a lesion surrounded by a hyperpigmented edge and yellowish colour appearing at the centre characteristic of the atrophic scar. The rest of the fundus and vitreous in both eyes were normal. Enzyme-linked immunosorbent assay (ELISA) of serum for anti-toxoplasma IgG antibodies showed high titers, more than 1.11. ELISA for human immunodeficiency virus was negative. Our patient could not receive active treatment for the right eye macular scar as the visual loss is irreversible. The patient was advised a regular six-monthly follow-up to detect any recurrences.

**Figure 1:**
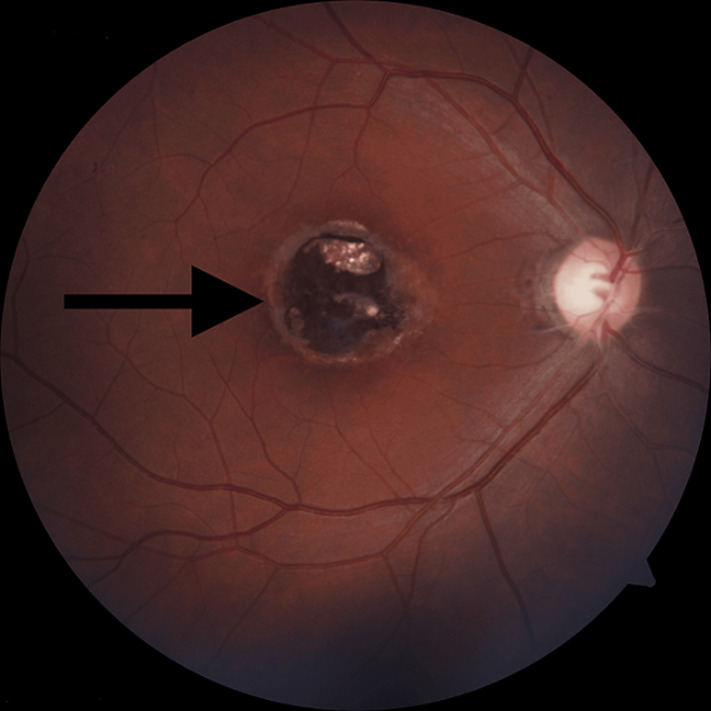
fundus photograph showing a chorioretinal scar in the right macular area, which was secondary to toxoplasmosis characterised by the presence of a lesion surrounded by a hyperpigmented edge and yellowish colour appearing at the centre characteristic of the atrophic scar

